# Healthy aging in humanitarian crises: Addressing the neglect of older adults in nutrition policies

**DOI:** 10.1016/j.tjfa.2026.100215

**Published:** 2026-10-13

**Authors:** Abdisalan Abdikarin Mohamed, Abdikadir Ahmed Hassan, Fartun Ali Haji Aden, Mohamed Daud Mohamed, Yakub Burhan Abdullahi

**Affiliations:** a Somali National University Faculty of Health Science and Tropical Medicine SOMALIA, Somalia; b Faculty of Health Science, Salaam University, Mogadishu, Somalia; c Center for Health Research and Innovation, Somali National University, Mogadishu, Somalia; d Federal Ministry of Health, Somalia; e Benaadir Research, Consultancy and Evaluation Center, Mogadishu, Somalia

Dear Editor,

Humanitarian nutrition responses often identify children and pregnant or lactating women as priority groups, while the nutritional needs of older adults receive less systematic attention. This is a consequential gap: food insecurity, illness, poor appetite, and reduced mobility can interact with malnutrition, sarcopenia, and frailty to threaten an older person’s ability to remain independent [[1], [2], [3]]. The question for humanitarian policy is therefore practical: how can programmes identify and support older adults before preventable nutritional decline becomes disability?

Existing guidance shows both the opportunity and the implementation gap. Standards for including older people in humanitarian nutrition responses already exist, yet HelpAge International reports that older people may be overlooked in needs assessments, face barriers to reaching assistance, or receive support that does not account for their nutritional needs [4]. Without nutrition data disaggregated by age and disability, planners cannot readily determine whether older adults are being reached or whether the assistance provided meets their needs. A general food distribution may also be difficult to use for someone who cannot travel to a distribution point, carry a ration, chew the available food, or prepare it without help [4]. These are identifiable programme design and access problems, not simply a lack of food.

Somalia illustrates why this distinction matters. The country’s 2026 humanitarian response plan estimates that 4.8 million people need assistance amid drought, conflict, and disease outbreaks [5]. UNICEF’s 2026 appeal identifies older people within the affected population, but its prominent nutrition estimates and targets focus on children [6]. This does not establish that older Somalis receive no assistance; it shows why age-specific nutrition assessment, service targets, and reporting should be visible alongside existing priorities.

A feasible response has three parts. First, nutrition clusters should report needs, reach, and outcomes by age, sex, and disability, including for older adults [4]. Second, community and facility teams should ask older people about recent weight loss, appetite, food access, and difficulties eating, then arrange fuller nutritional and functional assessment when concerns are identified [2,3]. Third, programmes should adapt food delivery and support to assessed needs: accessible distribution or home delivery where necessary, help with food preparation, dietary advice, and oral nutritional supplementation for people affected by undernutrition when appropriate [4,7]. Frailty assessment can guide broader care, while nutrition support should remain tailored to health status and individual goals [1,7].

The contribution needed now is to translate existing guidance into measurable humanitarian practice. Recording whether older adults are assessed, reached, and supported would make their needs harder to overlook and would connect emergency nutrition assistance to the preservation of function and independence.

## Funding

No funding was received for this work.

## Ethics approval

Not applicable; this letter did not involve research with human participants or animals.

## Declaration of generative AI

The authors used generative AI solely for limited language refinement. The authors reviewed and approved the final manuscript and take full responsibility for its content.

## CRediT authorship contribution statement

**Abdisalan Abdikarin Mohamed:** Writing – original draft, Visualization. **Abdikadir Ahmed Hassan:** Writing – review & editing. **Fartun Ali Haji Aden:** Methodology, Conceptualization. **Mohamed Daud Mohamed:** Validation. **Yakub Burhan Abdullahi:** Supervision.

## Declaration of competing interest

The authors declare no competing interests.
